# Self-reported changes in sexual behaviors and HIV prevention services utilization among gay, bisexual and other men who have sex with men after entering the post-pandemic era: tales of two Chinese cities with different pathways of “resume normal”

**DOI:** 10.1186/s12889-025-22756-7

**Published:** 2025-04-21

**Authors:** Xinge Li, Siyu Chen, Zheng Zhang, Shen Ge, Lijuan Wang, Xinyue Chen, Fuk-yuen Yu, Yuan Fang, Zihuang Chen, Zhennan Li, Fenghua Sun, Yingjie Liu, Zixin Wang

**Affiliations:** 1 Beijing Chaoyang District Center for Disease Control and Prevention, Beijing, China; 2 Centre for Health Behaviours Research, JC School of Public Health and Primary Care, Chinese University of Hong Kong, Hong Kong, Hong Kong SAR China; 3 Department of Health and Physical Education, the Education University of Hong Kong, Hong Kong , Hong Kong SAR China; 4 Danlan Goodness, Beijing, China; 5 Hongshiliu Goodness, Beijing, China

**Keywords:** Self-reported changes, Sexual behaviors, HIV prevention services, Resume normal, Post-pandemic era, Gay, bisexual and other men who have sex with men, China

## Abstract

**Background:**

Relaxing Coronavirus Disease 2019 (COVID-19) control measures and “resume normal” might have direct impacts on sexual behaviors and utilization of HIV prevention and sexual health services among gay, bisexual and other men who have sex with men (GBMSM). To address the knowledge gaps, this study aimed to compare self-reported changes in sexual risk behaviors and HIV service utilization among GBMSM in the post-pandemic era between Beijing and Hong Kong. In addition, the determinants of self-reported increase in condomless anal sex (CAS) were investigated among GBMSM in Beijing or Hong Kong.

**Methods:**

This cross-sectional study was conducted between November 2023 and March 2024. Participants were Chinese males aged ≥ 18 years who had anal sex with at least one man in the past six months recruited through multiple sources. Eligible GBMSM completed a telephone interview. A multiple logistic regression model was then fitted.

**Results:**

A total of 524 GBMSM in Beijing and 613 in Hong Kong completed the telephone interview. After adjusting for background characteristics with between-city differences, more GBMSM in Beijing self-reported an increase in the frequency of CAS with men (23.7% versus 7.8%, *p* < 0.001), anal sex with regular male sex partners (30.5% versus 15.5%, *p* < 0.001) and non-regular male sex partners (27.5% versus 16.0%, *p* < 0.001), seeking male partners online (28.8% versus 18.9%, *p* < 0.001), sexualized drug use (SDU) (9.4% versus 2.3%, *p* < 0.001), and using HIV testing (31.5% versus 11.7%, *p* < 0.001), pre-exposure prophylaxis (PrEP) (18.9% versus 3.6%, *p* < 0.001) and other HIV prevention services (20.2% versus 10.1%, *p* < 0.001). GBMSM in both cities who self-reported increases in the frequency of anal sex with regular and non-regular male sex partners, having sex with men coming from other cities, seeking male partners online, utilization of SDU, HIV testing, PrEP and other HIV prevention services were more likely to report an increase in CAS.

**Conclusions:**

More GBMSM in Beijing than their counterparts in Hong Kong reported increases in the frequency of sexual risk behaviors and HIV prevention services utilization when comparing their current situation with the time during COVID-19. Our findings highlighted the importance of strengthening HIV prevention in GBMSM in the post-pandemic era.

## Background

Globally, HIV remains a serious public health threat for gay, bisexual and other men who have sex with men (GBMSM). The overall HIV prevalence among GBMSM in China was 5.7% in 2018 [1], with an HIV incidence of 4.9 per 100 person-year [2]. This study was conducted in Beijing and Hong Kong. Beijing is the capital of the People’s Republic of China with over 22 million residents. Hong Kong is a special administrative region of the People’s Republic of China with 7.4 million residents. Hong Kong maintains a separated governing and economic system from that of mainland China under the principle of one country and two systems. The HIV prevalence among GBMSM was 10.6% in Beijing and 6.54% in Hong Kong [3–5], with an estimated HIV incidence of 3.5 per 100 person-year in Beijing and 1.0 per 100 person-year in Hong Kong [5, 6], respectively.

The Coronavirus Disease 2019 (COVID-19) pandemic and its control measures have had direct impacts on sexual behaviors and the utilization of HIV prevention and sexual health services among GBMSM. Previous studies have shown that GBMSM had reduced amount of sexual behaviors and number of sex partners during the COVID-19 period [7–10]. They have also adopted strategies to reduce the risk of COVID-19 via sexual behaviors, such as avoiding sex with casual partners or group sex parties [7–10]. A study conducted in mainland China showed that the prevalence of condomless anal sex (CAS) and sexualized drug use (SDU) among GBMSM declined after COVID-19 outbreak [11]. In Hong Kong, GBMSM reported significantly fewer male sex partners during the COVID-19 period than before COVID-19. However, higher levels of CAS have been observed with different types of partners [10]. The number of HIV tests performed in Japan and Australia decreased significantly after the COVID-19 outbreak [9, 12]. In the United States, about 20% of GBMSM had decreased access to HIV testing services after the COVID-19 outbreak [7]. A similar situation was observed in mainland China and Hong Kong [11, 13, 14]. A significant decline (about 20%) in the HIV testing rate was observed among GBMSM in mainland China during the COVID-19 period, as compared to the time before COVID-19 [11]. In addition, a significant decline in the use and adherence of pre-exposure prophylaxis (PrEP) was also observed among GBMSM in mainland China, due to increased difficulties in accessing medication and HIV testing services, and decreased sexual risk behaviors [15]. In Hong Kong, some governmental testing facilities were suspended during the pandemic as the government reallocated resources to focus on COVID-19 control [16]. To further worsen the situation, some community-based organizations in Hong Kong suspended physical outreach and education programs for GBMSM [16]. As a result, 56.8% of GBMSM in Hong Kong encountered difficulties in accessing facility-based HIV testing services during the COVID-19 period [14].

Similar to most parts of the world, both mainland China and Hong Kong resumed normal by lifting strict COVID-19 control measures in 2022. It is likely that the levels of sexual risk behaviors would increase among GBMSM after strict control measures were lifted. A previous study observed a 10% increase in CAS, multiple sex partnerships, and SDU among GBMSM in mainland China when the COVID-19 situation was under initial control [11]. However, there are concerns that the utilization of HIV prevention services may not increase after resuming normal, as the aforementioned study did not observe significant changes in the HIV testing rate among GBMSM after the initial control of COVID-19 [11]. Increasing sexual risk behaviors with no changes in HIV prevention services utilization may lead to potential HIV outbreaks. There was a lack of data on the changes in HIV-related behaviors and service utilization among GBMSM in the post-pandemic era.

To address this knowledge gap, this study aimed to compare the self-reported changes in sexual behaviors (i.e., anal sex with different types of male partners, CAS, and SDU) and HIV preventive services utilization (i.e., HIV testing, PrEP, and other services) among GBMSM in the post-pandemic era between Beijing and Hong Kong. In addition, determinants of self-reported increase in the frequency of CAS, one of the most important risk factors for HIV acquisition among GBMSM in China [17], were also investigated among GBMSM in Beijing or Hong Kong. Potential determinants considered in this study included background characteristics, self-reported increases in other HIV-related behaviors, and changes in perceptions related to HIV, condom use and COVID-19.

## Methods

### Study design

**Fig. 1:**
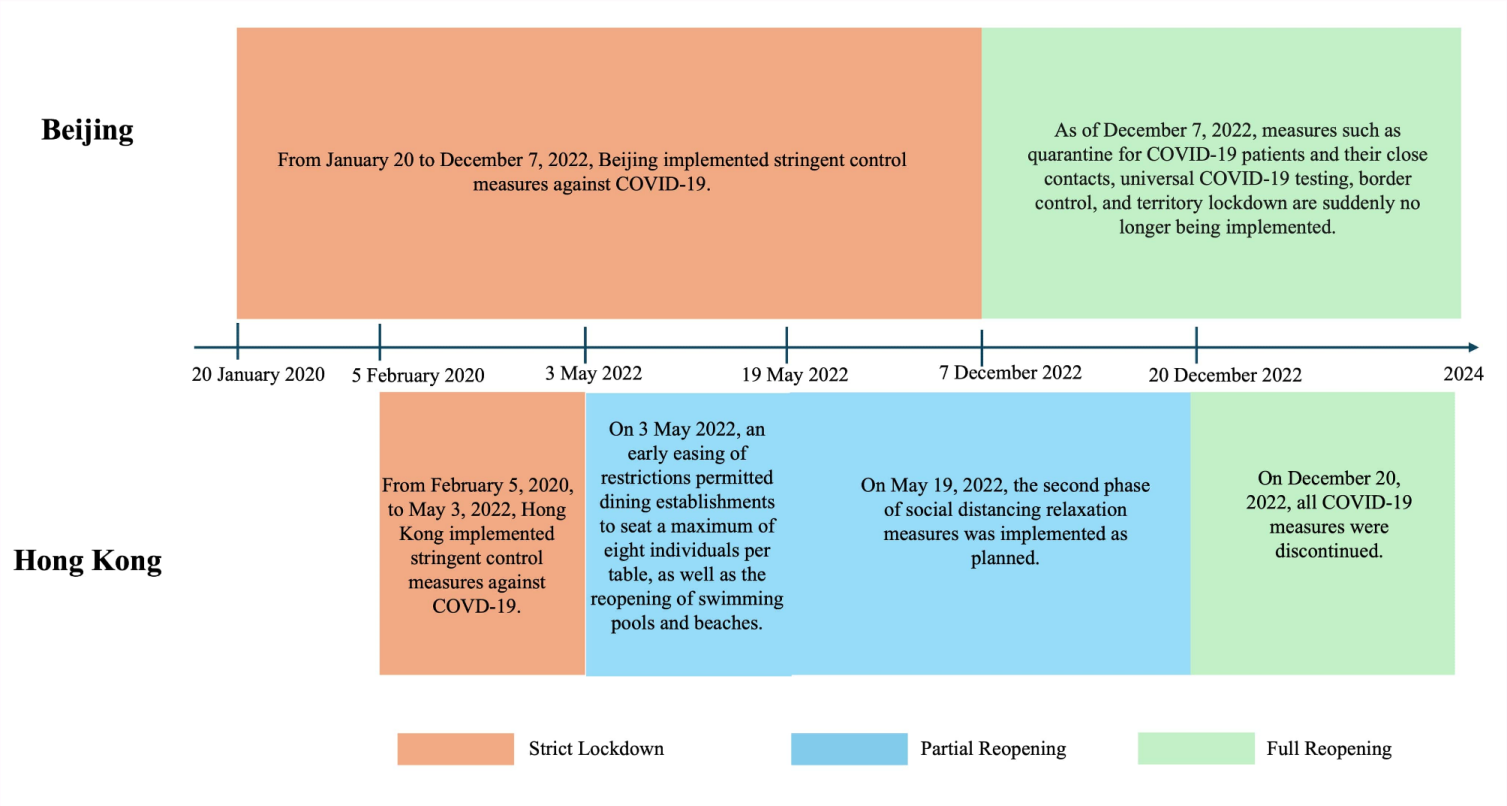
The differences in COVID-19 control measures between Beijing and Hong Kong between 2020 and 2024

A cross-sectional survey was conducted among GBMSM in Beijing and Hong Kong between November 2023 and March 2024. The differences in COVID-19 control measures between Beijing and Hong Kong between 2020 and 2024 were illustrated in Fig. 1.

### Participants and data collection

The inclusion criteria were as follows: (1) Chinese males aged ≥ 18 years, and (2) having had anal sex with at least one man in the past six months. Recruitment and data collection methods were similar between the two study sites. In both cities, upon approval from the owners, trained and experienced fieldworkers approached potential participants in gay bars at different time slots on weekdays and weekends. On sites, the fieldworkers briefed potential participants about the study and gave them an information sheet. Online recruitment was also performed in both cities by posting study advertisements on popular gay websites (in Hong Kong only), social media platforms such as Facebook (in Hong Kong only) or Weibo/WeChat (in Beijing only), and partner-seeking mobile applications frequently used by local GBMSM (BlueD in Beijing and Grindr in Hong Kong). Facebook is the most popular social media platform in Hong Kong [18], and Grindr is the most widely used gay dating application in the same city [19]. However, these two online platforms are not available in mainland China, including Beijing. Weibo and WeChat are the two most commonly used social media platforms in mainland China. They are very similar to Facebook in where users can connect to others by sharing videos, pictures, text and live streams. BlueD is the most widely used gay dating application in mainland China, with features and functions very similar to those of Grindr [20]. Therefore, we chose Weibo/WeChat and BlueD to facilitate the recruitment of GBMSM in Beijing. Potential participants who were interested in the study contacted the research team through private messaging, telephone, WhatsApp/WeChat, or email. Recruitment was supplemented by referral from peers and community-based organizations. Participants were screened for study eligibility and informed of their guaranteed anonymity and right to quit at any time, and their refusal would not have any consequences. Two approaches were used to maintain the participants’ anonymity. First, verbal instead of written consent was collected by fieldworkers. Second, we did not collect the participant’s name or other identifiable personal information. Multiple contact methods were obtained (i.e., mobile phone number and social media account) to schedule telephone interviews. Participants’ contact information was kept separate from the questionnaire and would not appear on the questionnaire or in the dataset for analysis. The interviews were administered by trained and experienced interviewers in Mandarin in Beijing and Cantonese in Hong Kong, which is the dominant spoken language of the city, respectively. The questionnaire used by the interviewers was written in simplified Chinese in Beijing and in traditional Chinese in Hong Kong. The contents were the same and comparability was ensured. During the telephone interview, the trained interview read out the questions and their response categories to the participants, and then the participants responded with a choice. Our team is experienced in conducting telephone surveys of GBMSM. The interviewers are familiar with the culture of GBMSM. The team completed several telephone surveys among GBMSM related to sexual behaviors and/or HIV-related prevention service utilization [21–23]. Compared to the self-administered online survey, the telephone surveys had a higher completion rate as rapport was established between participants and the interviewers. The telephone surveys offered a relatively high flexibility, as we were able to ask complicated or open-ended questions. It is also easier to exercise quality control when telephone interviews are used. Upon completion of the 30-minutes telephone interviews, an HK$50 (US$6.4) supermarket coupon was mailed to participants in Hong Kong. Participants in Beijing received an e-coupon of ¥20 (US$3.0) after completing the interview. The amount of incentive and the way to dispatch the incentive were similar to those in studies targeting GBMSM in mainland China and Hong Kong [10, 11, 21]. Ethical approval was obtained from the Survey and Behavioral Research Ethics Committee of the Chinese University of Hong Kong (reference no.: SBRE-22-0488).

### Sample size planning

We aimed to recruit 500 participants in each city. Previous studies showed that about 20% of GBMSM in mainland China reported a decline in CAS after the COVID-19 outbreak [11]. Another study suggested that the prevalence of CAS among GBMSM in the post-pandemic era was similar to that reported in the time before COVID-19 [24]. Therefore, we assumed a 20% increase in CAS in our sample after entering post-pandemic (i.e., comparing their current situation with that during the COVID-19 pandemic). Such sample size could detect a smallest difference of 6.6% in the dependent variable between participants in Beijing and Hong Kong, given a statistical power of 0.80 and an alpha value of 0.05 (two-sided) (PASS 11.0, NCSS LLC).

## Measurements

### Development of the questionnaire

A panel consisting of several public health researchers, behavioral health scientists, health psychologists, staff of community-based organizations, and the Center for Disease Control and Prevention, and local GBMSM was formed to develop the questionnaire. A pilot study was conducted among 20 GBMSM, 10 in Beijing and the other 10 in Hong Kong, to examine the clarity and readability of the questionnaire. All participants in the pilot study found the questions easy to understand and the length acceptable. The panel finalized the questionnaire for the actual survey. These 20 GBMSM were not counted in the target sample size.

### Background characteristics

Participants were asked to report their sociodemographic characteristics (i.e., age, current relationship status, education level, employment status, and monthly personal income), sexual orientation, and HIV sero-status. In addition, information on the history of COVID-19 infection confirmed by rapid antigen testing and/or nucleic acid amplification tests and COVID-19 vaccination status was collected.

### HIV-related behaviors in the past six months

Participants reported the number of regular and non-regular male sex partners with anal sex, CAS with partners in each category, and SDU in the past six months. In line with previous studies, regular male sex partners (RP) was defined as their stable boyfriends or lovers, while non-regular male sex partners (NRP) was defined as casual sex partners or male sex workers [21, 25]. SDU refers to the use of any of the following psychoactive substances before or during sexual sex, including ketamine, methamphetamine, cocaine, cannabis, ecstasy, Dormicum/Halcion/Erimin 5/non-prescription hypnotic drugs, heroin, cough suppressant (not for curing cough), amyl nitrite (popper), GHB/ GBL, 5-methoxy-N, N-diisopropyltryptamine (Foxy), and mephedrone [21, 25]. Utilization of HIV testing, PrEP, testing for other sexually transmitted infections (STI), and other HIV prevention services (i.e., receiving condoms or lubricants, health education, pamphlets, or seminars/workshops) was also documented.

### Self-reported changes in HIV-related behaviors and perceptions

In this study, the COVID-19 period refers to the time between December 2019 (COVID-19 outbreak in China) and December 2022 (all strict COVID-19 control measures were lifted in both mainland China and Hong Kong) [26–28]. The measurements of self-reported changes in HIV-related behaviors and perceptions were shown in Table 1. Participants were asked whether they increased the frequency of the following behaviors by comparing their current situation with the COVID-19 period, including anal sex with RP and NRP, CAS with men, SDU, having sex with men coming from other cities, seeking male partners via Internet or gay social networking applications, and utilization of HIV testing and other HIV prevention services (i.e., receiving condoms or lubricant, health education, pamphlets or seminars/workshops). Participants who self-reported to be HIV negative or unknown sero-status answered two additional questions measuring self-reported increase in the use of HIV testing and PrEP (response categories: 0 = no & 1 = yes). Five other items measured changes in perceptions comparing their current situation versus the time during the COVID-19 period, including perceived increase in the overall chance of HIV transmission, risk of contracting COVID-19, fear of COVID-19, perceived decrease in the difficulties in seeking male sex partners, and self-efficacy of consistent condom use during anal sex (response categories: 0 = no & 1 = yes).

**Table 1:** Measurements of self-reported changes in HIV-related behaviors and perceptions

Items	Response categories
**Self-reported changes in HIV-related behaviors**	
Did you increase the frequency of the following behaviors when comparing your current situation with the COVID-19 period?	0 = No, 1 = Yes
Condomless anal sex with men	
Anal sex with regular male sex partners	
Anal sex with non-regular male sex partners	
Sex with GBMSM in other cities	
Seeking male partners via Internet, gay social networking applications and geospatial dating applications	
Sexualized drug use	
HIV testing uptake	
Using other HIV prevention services (e.g., STI testing, seminar/workshop, visiting NGO)	
**Self-reported changes in perceptions**	
Your overall chance of HIV transmission increased when comparing your current situation with the COVID-19 period	0 = No, 1 = Yes
Your difficulties in seeking male sex partners decreased when comparing your current situation with the COVID-19 period	0 = No, 1 = Yes
Your self-efficacy of consistent condom use during anal sex decreased when comparing your current situation with the COVID-19 period	0 = No, 1 = Yes
Your risk of contrasting COVID-19 during sexual behaviors increased when comparing your current situation with the COVID-19 period	0 = No, 1 = Yes
Your fear of COVID-19 increased when comparing your current situation with the COVID-19 period	0 = No, 1 = Yes

### Statistical analysis

We followed the analytical plan of some published papers on similar topics [23, 29–31]. The frequency distribution of all studied variables was presented. Differences in background characteristics between participants in Beijing and Hong Kong were compared using the chi-square tests. The between-city differences in HIV-related behaviors and perceived changes in behaviors and perceptions were then compared using logistic regression models, after controlling for background characteristics with significant between-city differences. Perceived increase in CAS with men was used as the dependent variable. Bivariate logistic regression models were fitted to assess the significance between background characteristics and the dependent variable among participants in each city. Each logistic regression model contained only one independent variable of interest (i.e., perceived change in another HIV-related behavior) and covariates (i.e., significant background characteristics). Crude odds ratios (OR), adjusted OR (AOR), and their 95% confidence intervals (CI) were obtained. SPSS 26.0 (IBM Corp., Armonk, NY, USA) was used for data analysis, and *p* < 0.05 was the level of statistically significance.

## Results

### Background characteristics of the participants

The research team approached 602 and 713 eligible GBMSM in Beijing and Hong Kong, 78 and 100 refused to participate in the study due to time or other tangible reasons, and 524 and 613 completed the telephone interview, respectively. The response rates were similar between the two cities (87% in Beijing and 86% in Hong Kong). Over half of the participants in Beijing and Hong Kong were under 35 years old (65.9% & 56.1%), currently single (70.6% & 82.1%), had attained tertiary education (86.8% & 83.8%), had full-time employment (79.6% & 77.3%), and had a monthly personal income no lower than the median income level of the city (56.3% & 68.8%). The majority of the participants had a history of confirmed COVID-19 infection (84.2% & 82.2%), and had received at least three doses of COVID-19 vaccination (77.3% & 81.5%). A total of 32 (6.1%) and 8 (1.3%) participants self-reported as HIV-positive in Beijing and Hong Kong, respectively. Compared to participants in Beijing, participants in Hong Kong were more likely to be older, currently single, have a monthly personal income no lower than the median income level of the city, self-reported as HIV-negative or of unknown sero-status, and had received more than three doses of COVID-19 vaccination (*p* < 0.001 in all comparisons) (Table 2).

**Table 2:** Background characteristics of GBMSM in Beijing and Hong Kong, China

	Beijing (*n* = 524)	Hong Kong (*n* = 613)	*p* value
	*n* (%)	*n* (%)	
Age group (years)			
18–24	67 (12.8)	72 (11.8)	
25–34	278 (53.1)	271 (44.3)	
35–44	138 (26.3)	170 (27.8)	
45 or above	41 (7.8)	99 (16.2)	< 0.001^**^
Current relationship status			
Currently single	370 (70.6)	503 (82.1)	
Married or cohabited with a man	119 (22.7)	105 (17.1)	
Married or cohabited with a woman	35 (6.7)	5 (0.5)	< 0.001^**^
Highest education level attained			
Senior high or below	69 (13.2)	99 (16.2)	
College or above	455 (86.8)	514 (83.8)	0.16
Having a full-time employment			
Yes	417 (79.6)	474 (77.3)	
No	107 (20.4)	139 (22.7)	0.36
Monthly personal income			
Below median income level ^1^	194 (37.0)	187 (30.5)	
Median income or above	295 (56.3)	422 (68.8)	
Refuse to disclose	35 (6.7)	4 (0.7)	< 0.001^**^
Sexual orientation			
Gay	405 (77.3)	549 (89.6)	
Bisexual	81 (15.5)	59 (9.6)	
Heterosexual	7 (1.3)	2 (0.3)	
Uncertain	31 (5.9)	3 (0.5)	< 0.001^**^
Self-reported to be HIV positive			
No	492 (93.9)	605 (98.7)	
Yes	32 (6.1)	8 (1.3)	< 0.001^**^
History of confirmed COVID-19 infection			
No	83 (15.8)	109 (17.7)	
Yes	441 (84.2)	504 (82.2)	0.38
Number of doses of COVID-19 vaccination received by the participants			
0	22 (4.2)	19 (3.0)	
1	8 (1.5)	9 (1.5)	
2	89 (17.0)	86 (14.0)	
3	375 (71.6)	419 (68.4)	
4	23 (4.4)	76 (12.4)	
5	7 (1.3)	4 (0.7)	< 0.001^**^

^*^p < 0.05, ^**^p < 0.01

^1^ Median income of the residents was ¥7,000 (US$980) per month in Beijing and HK$20,000 (US$2,564) per month in Hong Kong

### Difference in HIV-related behaviors between Beijing and Hong Kong

After adjusting for background characteristics with between-city differences, GBMSM in Hong Kong had a higher prevalence of CAS (56.0% versus 44.1%, *p* < 0.001) but a lower prevalence of SDU (9.3% versus 18.3%, *p* < 0.001), as compared to their counterparts in Beijing. The proportion of participants who had anal sex with RP (70.6% versus 74.2%, *p* = 0.18), NRP (51.0% versus 46.3%, *p* = 0.16), and multiple sex partnerships (55.7% versus 50.6%, *p* = 0.12) was similar between cities. Compared to GBMSM in Hong Kong, significantly more participants in Beijing used HIV testing (80.2% versus 46.3%, *p* < 0.001), PrEP (18.3% versus 14.3%, *p* = 0.01), STI testing (63.7% versus 38.8%, *p* < 0.001), and other HIV-related prevention services (59.9% versus 44.5%, *p* < 0.001) in the past six months (Table 3).

**Table 3:** Sexual behaviors and HIV-related services utilization in the past six months

	Beijing (*n* = 524)	Hong Kong (*n* = 613)	Unadjusted *p* value	Adjusted *p* value ^1^
	*n* (%)	*n* (%)		
**Sexual behaviors in the past six months**				
Anal sex with regular male sex partners				
No	154 (29.4)	158 (25.8)		
Yes	370 (70.6)	455 (74.2)	0.17	0.18
Anal sex with non-regular male sex partners or male sex workers				
No	257 (49.0)	329 (53.7)		
Yes	267 (51.0)	284 (46.3)	0.12	0.16
Condomless anal sex with men				
No	293 (55.9)	270 (44.0)		
Yes	231 (44.1)	343 (56.0)	< 0.001	< 0.001^**^
Multiple male sex partnership				
No	232 (44.3)	303 (49.4)		
Yes	292 (55.7)	310 (50.6)	0.08	0.12
Sexualized drug use				
No	428 (81.7)	556 (90.7)		
Yes	96 (18.3)	57 (9.3)	< 0.001	< 0.001^**^
**HIV-related services utilization in the past six months**				
Use of any types of HIV testing				
No	72 (13.7)	321 (52.3)		
Yes	420 (80.2)	284 (46.3)		
Not applicable	32 (6.1)	8 (1.3)	< 0.001	< 0.001^**^
Use of pre-exposure prophylaxis				
No	396 (75.6)	517 (84.3)		
Yes	96 (18.3)	88 (14.3)		
Not applicable	32 (6.1)	8 (1.3)	0.03	0.01^*^
Testing for other sexually transmitted infections				
No	190 (36.3)	375 (61.2)		
Yes	334 (63.7)	238 (38.8)	< 0.001	< 0.001^**^
Use of other HIV-related services				
No	210 (40.1)	340 (55.5)		
Yes	314 (59.9)	273 (44.5)	< 0.001	< 0.001^**^

^*^p < 0.05, ^**^p < 0.01

^1^ Adjusted *p* values: *p* values obtained from multivariable logistic regression after adjusting for background characteristics with significant between-group difference in Table 2 (age group, current relationship status, monthly personal income, sexual orientation, and number of doses of COVID-19 vaccination received by the participants)

### Self-reported changes in behaviors

After adjusting for background characteristics with between-city differences, more participants in Beijing than in Hong Kong reported an increase in the frequency of CAS with men (23.7% versus 7.8%, *p* < 0.001), SDU (9.4% versus 2.3%, *p* < 0.001), and PrEP (18.9% versus 3.6%, *p* < 0.001). Compared to GBMSM in Hong Kong, more participants in Beijing reported increases in the frequency of anal sex with RP (30.5% versus 15.5%, *p* < 0.001) and NRP (27.5% versus 16.0%, *p* < 0.001), seeking male partners online (28.8% versus 18.9%, *p* < 0.001), using HIV testing (31.5% versus 11.7%, *p* < 0.001), and other HIV prevention services (20.2% versus 10.1%, *p* < 0.001). Between-city comparisons of the changes in perceptions are shown in Table 4.

**Table 4:** Self-reported changes in HIV-related behaviors and perceptions comparing their current situation with the COVID-19 period

	Beijing (*n* = 524)	Hong Kong (*n* = 613)	Unadjusted *p* value	Adjusted *p* value ^1^
	*n* (%)	*n* (%)		
**Dependent variable**				
Self-reported increase in frequency of condomless anal sex with men comparing their current situation with the COVID-19 period				
No	400 (76.3)	565 (92.2)		
Yes	124 (23.7)	48 (7.8)	< 0.001	< 0.001^**^
**Independent variables: Self-reported increases in other HIV-related behaviors comparing their current situation with the COVID-19 period**				
Self-reported increase in frequency of anal sex with regular male sex partners				
No	364 (69.5)	518 (84.5)		
Yes	160 (30.5)	95 (15.5)	< 0.001	< 0.001^**^
Self-reported increase in frequency of anal sex with non-regular male sex partners				
No	380 (72.5)	515 (84.0)		
Yes	144 (27.5)	98 (16.0)	< 0.001	< 0.001^**^
Self-reported increase in frequency of having sex with GBMSM in other cities				
No	475 (90.6)	538 (87.8)		
Yes	49 (9.4)	75 (12.2)	0.12	0.37
Self-reported increase in frequency of seeking male partners via Internet, gay social networking applications and geospatial dating applications				
No	373 (71.2)	497 (81.1)		
Yes	151 (28.8)	116 (18.9)	< 0.001	< 0.001^**^
Self-reported increase in frequency of sexualized drug use				
No	475 (90.6)	599 (97.7)		
Yes	49 (9.4)	14 (2.3)	< 0.001	< 0.001^**^
Self-reported increase in frequency of HIV testing				
No/not applicable	359 (68.5)	541 (88.3)		
Yes	165 (31.5)	72 (11.7)	< 0.001	< 0.001^**^
Self-reported increase in frequency of using pre-exposure prophylaxis (PrEP)				
No/not applicable	425 (81.1)	591 (96.1)		
Yes	99 (18.9)	22 (3.6)	< 0.001	< 0.001^**^
Self-reported increase in frequency of using other HIV prevention services (e.g., STI testing, seminar/workshop, visiting NGO)				
No	418 (79.8)	551 (89.9)		
Yes	106 (20.2)	62 (10.1)	< 0.001	< 0.001^**^
**Independent variables: Changes in perceptions comparing their current situation with the COVID-19 period**				
Perceived increase in overall chance of HIV transmission				
No	365 (69.7)	523 (85.3)		
Yes	159 (30.3)	90 (14.7)	< 0.001	< 0.001^**^
Perceived decrease in difficulties in seeking male sex partners				
No	406 (77.5)	457 (74.6)		
Yes	118 (22.5)	156 (25.4)	0.25	0.84
Perceived decrease in self-efficacy of consistent condom use during anal sex				
No	446 (85.1)	600 (97.9)		
Yes	78 (14.9)	13 (2.1)	< 0.001	< 0.001^**^
Perceived increase in risk of contrasting COVID-19 during sexual behaviors				
No	423 (80.7)	509 (83.0)		
Yes	101 (19.3)	104 (17.0)	0.31	0.11
Increase in fear of COVID-19				
No	437 (83.4)	583 (95.1)		
Yes	87 (16.6)	30 (4.9)	< 0.001	< 0.001^**^

^*^p < 0.05, ^**^p < 0.01

^1^ Adjusted *p* values: *p* values obtained from multivariable ordinal regression after adjusting for background characteristics with significant between-group difference in Table 2 (age group, current relationship status, monthly personal income, sexual orientation, and number of doses of COVID-19 vaccination received by the participants)

### Factors associated with increased CAS

In Hong Kong, participants aged 25–34 years were less likely to report an increase in CAS, as compared to those aged 18–24 years (OR: 0.32, 95%CI: 0.13, 0.80, *p* = 0.02) (Table 5).

**Table 5:** Associations between background characteristics and self-reported increase in frequency of condomless anal sex with men

	Model 1: Beijing (*n* = 524)			Model 2: Hong Kong (*n* = 613)		
	OR	95% CI	*p* value	OR	95% CI	*p* value
Age group (years)						
18–24	Reference			Reference		
25–34	0.98	0.51; 1.85	0.94	0.32	0.13; 0.80	0.02^*^
35–44	1.22	0.61; 2.44	0.57	0.53	0.21; 1.32	0.18
45 or above	1.43	0.59; 3.47	0.42	1.25	0.51; 3.04	0.62
Current relationship status						
Currently single	Reference			Reference		
Married or cohabited with a man	1.22	0.76; 1.96	0.42	0.81	0.35; 0.85	0.61
Married or cohabited with a woman	1.38	0.64; 3.00	0.41	N.A.	N.A.	N.A.
Highest education level attained						
Senior high or below	Reference			Reference		
College or above	0.94	0.52; 1.70	0.84	0.96	0.44; 2.12	0.92
Having a full-time employment						
Yes	Reference			Reference		
No	0.92	0.55; 1.52	0.74	1.62	0.85; 3.07	0.14
Monthly personal income						
Below median income level	Reference			Reference		
Median income or above	1.18	0.77; 1.81	0.44	0.65	0.36; 1.20	0.17
Refuse to disclose	0.44	0.15; 1.31	0.14	N.A.	N.A.	N.A.
Sexual orientation						
Gay	Reference			Reference		
Bisexual	0.67	0.36; 1.22	0.19	0.60	0.18, 1.99	0.40
Heterosexual	0.49	0.06, 4.11	0.51	N.A.	N.A.	N.A.
Uncertain	0.56	0.21; 1.51	0.25	N.A.	N.A.	N.A.
Self-reported to be HIV positive						
No	Reference			Reference		
Yes	0.58	0.22; 1.54	0.28	N.A.	N.A.	N.A.
History of confirmed COVID-19 infection						
No	Reference			Reference		
Yes	0.97	0.56; 1.68	0.92	1.09	0.49; 2.40	0.83
Number of doses of COVID-19 vaccination received by the participants						
0	Reference			Reference		
1	0.64	0.06; 6.80	0.71	0.67	0.06; 7.48	0.74
2	1.76	0.54; 5.71	0.35	0.47	0.11; 2.03	0.31
3	1.38	0.46; 4.18	0.57	0.40	0.11; 1.44	0.16
4	1.25	0.29; 5.43	0.77	0.54	0.13; 2.33	0.41
5	0.75	0.07; 8.09	0.81	1.78	0.14; 23.40	0.66

^*^p < 0.05, ^**^p < 0.01

OR: crude odds ratios

CI: confidence interval

After adjusting for age group, participants in both cities who self-reported increases in the frequency of anal sex with RP (AOR: 20.49 & 8.70, *p* < 0.001) and NRP (AOR: 13.26 & 4.39, *p* < 0.001), having sex with men coming from other cities (AOR: 12.05 & 3.02, *p* < 0.001 & *p* = 0.003), seeking male partners online (AOR: 9.00 & 3.15, *p* < 0.001), SDU (AOR: 29.32 & 17.13, *p* < 0.001), and utilization of HIV testing (AOR: 5.18 & 3.30, *p* < 0.001 & *p* = 0.001), PrEP (AOR: 10.08 & 13.79, *p* < 0.001), and other HIV prevention services (AOR: 7.74 & 3.37, *p* < 0.001 & *p* = 0.002) were more likely to report an increase in CAS (Table 6).

**Table 6:** Factors associated with self-reported increase in frequency of condomless anal sex with men

	Model 1: Beijing (*n* = 524)			Model 2: Hong Kong (*n* = 613)		
	AOR	95% CI	*p* value	AOR	95% CI	*p* value
**Self-reported increases in other HIV-related behaviors comparing their current situation with the COVID-19 period**						
Self-reported increase in frequency of anal sex with regular male sex partners						
No	Reference			Reference		
Yes	20.49	12.28; 34.19	< 0.001^**^	8.70	4.60; 16.45	< 0.001^**^
Self-reported increase in frequency of anal sex with non-regular male sex partners (NRP)						
No	Reference			Reference		
Yes	13.26	8.26; 21.29	< 0.001^**^	4.39	2.33; 8.29	< 0.001^**^
Self-reported increase in frequency of having sex with GBMSM in other cities						
No	Reference			Reference		
Yes	12.05	6.12; 23.73	< 0.001^**^	3.02	1.46; 6.23	0.003^**^
Self-reported increase in frequency of seeking male partners via Internet, gay social networking applications and geospatial dating applications						
No	Reference			Reference		
Yes	9.00	5.72; 14.16	< 0.001^**^	3.15	1.64; 6.08	0.001^**^
Self-reported increase in frequency of sexualized drug use						
No	Reference			Reference		
Yes	29.32	12.67; 67.86	< 0.001^**^	17.13	5.44; 53.96	< 0.001^**^
Self-reported increase in frequency of HIV testing						
No/not applicable	Reference			Reference		
Yes	5.18	3.36; 7.99	< 0.001^**^	3.30	1.63; 6.70	0.001^**^
Self-reported increase in frequency of using pre-exposure prophylaxis (PrEP)						
No/not applicable	Reference			Reference		
Yes	10.08	6.16; 16.51	< 0.001^**^	13.79	5.21; 36.46	< 0.001^**^
Self-reported increase in frequency of using other HIV prevention services (e.g., STI testing, seminar/workshop, visiting NGO)						
No	Reference			Reference		
Yes	7.74	4.82; 12.44	< 0.001^**^	3.37	1.57; 7.17	0.002^**^
**Independent variables: Changes in perceptions comparing their current situation with the COVID-19 period**						
Perceived increase in overall chance of HIV transmission						
No	Reference			Reference		
Yes	6.67	4.30; 10.36	< 0.001^**^	3.23	1.66; 6.28	0.001^**^
Perceived decrease in difficulties in seeking male sex partners						
No	Reference			Reference		
Yes	5.80	3.69; 9.12	< 0.001^**^	1.82	0.97; 3.39	0.06
Perceived decrease in self-efficacy of consistent condom use during anal sex						
No	Reference			Reference		
Yes	9.15	5.37; 15.59	< 0.001^**^	17.52	5.32; 51.73	< 0.001^**^
Perceived increase in risk of contrasting COVID-19 during sexual behaviors						
No	Reference			Reference		
Yes	3.47	2.18; 5.52	< 0.001^**^	2.33	1.17; 4.61	0.02^*^
Increase in fear of COVID-19						
No	Reference			Reference		
Yes	6.54	3.99; 10.73	< 0.001^**^	1.46	0.41; 5.16	0.57

^*^p < 0.05, ^**^p < 0.01

OR: crude odds ratios

AOR: odds ratios adjusted for age group; each logistic regression model contained only one independent variable of interest (predictor) and one covariate (age group)

CI: confidence interval

Similar adjusted analysis showed that GBMSM in both cities who perceived an increase in overall chance of HIV infection (AOR: 6.67 & 3.23, *p* < 0.001) and risk of COVD-19 during sexual behaviors (AOR: 3.47 & 2.33, *p* < 0.001 & *p* = 0.02), and a decrease in self-efficacy of consistent condom use (AOR: 9.15 & 17.52, *p* < 0.001) were more likely to report an increase in CAS. Perceived a decrease in the difficulties in seeking male partners (AOR: 5.80, *p* < 0.001) and an increase in fear of COVID-19 (AOR: 6.54, *p* < 0.001) were positively correlated with self-reported increases in CAS among GBMSM in Beijing, but not among those in Hong Kong (Table 6). The most significant results in this study were summarized in Table 7.

**Table 7:** Summary of the most significant results

	Beijing (*n* = 524)	Hong Kong (*n* = 613)	Unadjusted *p* value	Adjusted *p* value
**Sexual behaviors and HIV-related services utilization in the past six months**,** Yes**				
Condomless anal sex with men	231 (44.1)	343 (56.0)	< 0.001	< 0.001^**^
Sexualized drug use	96 (18.3)	57 (9.3)	< 0.001	< 0.001^**^
HIV testing	420 (80.2)	284 (46.3)	< 0.001	< 0.001^**^
Use of pre-exposure prophylaxis (PrEP)	96 (18.3)	88 (14.3)	0.03	0.01^*^
**Self-reported changes in HIV-related behaviors comparing their current situation with the COVID-19 period**,** Yes**				
Self-reported increase in frequency of condomless anal sex with men	124 (23.7)	48 (7.8)	< 0.001	< 0.001^**^
Self-reported increase in frequency of sexualized drug use	49 (9.4)	14 (2.3)	< 0.001	< 0.001^**^
Self-reported increase in frequency of HIV testing	165 (31.5)	72 (11.7)	< 0.001	< 0.001^**^
Self-reported increase in frequency of using PrEP	99 (18.9)	22 (3.6)	< 0.001	< 0.001^**^

^*^p < 0.05, ^**^p < 0.01

OR: crude odds ratios

AOR: odds ratios adjusted for age group

CI: confidence interval

## Discussion

This was one of the first studies investigating the levels and self-reported changes in HIV-related behaviors among GBMSM after entering the post-pandemic era. This study addressed knowledge gaps with a relatively large sample size and a high response rate. Our findings provided a knowledge basis to inform HIV prevention service planning and the development of effective interventions for GBMSM in the post-pandemic era.

Among GBMSM in Hong Kong, the prevalence of CAS (56.0% versus 39.0%) and SDU (9.3% versus 7.3%) in the past six months observed in this study was higher than the figures reported in 2018–2019 [32, 33]. Similar trends were observed among GBMSM in Beijing, in which the levels of CAS (44.1% versus 35.8%) and multiple male sex partnerships (55.7% versus 37.2%) were higher than those reported before the COVID-19 outbreak [6]. Compared with the time before COVID-19, more GBMSM in both cities used PrEP. GBMSM might have stronger needs for PrEP due to the increase in sexual risk behaviors. In addition, the relaxation of control measures might have reduced GBMSM’s barriers to obtain PrEP. In mainland China (including Beijing), although the national program started to provide free PrEP to high-risk GBMSM in December 2021, lockdown and interruption of HIV testing services due to COVID-19 hindered GBMSM’s access to PrEP [34]. In Hong Kong, a large proportion of GBMSM PrEP users obtained medication from overseas clinics [35]. Travel restrictions and border controls made it difficult for them to travel abroad during the COVID-19 period. Although the HIV testing services resumed normal in both cities, the trends in HIV testing uptake differed between cities. In Beijing, the HIV testing uptake was higher than that before COVID-19 (80.2% versus 70.8%) [36]. However, in Hong Kong, the HIV testing uptake rate was similar to the figure reported before the COVID-19 outbreak (46.3% in the past six months versus 52.6% in the past year) [5]. More efforts are needed to enhance the HIV testing rate among GBMSM, especially in Hong Kong.

More GBMSM in Beijing than their counterparts in Hong Kong reported an increase in the frequency of sexual risk behaviors and HIV prevention services compared to their current situation during COVID-19. Some reasons might explain these between-city differences. Compared with Hong Kong, COVID-19 control measures were stricter and longer-lasting in Beijing. Previous studies have shown that stricter and longer-lasting COVID-19 restrictions enhanced stress, loneliness and other mental health challenges, which might be more likely to lead to increased risk behaviors as coping mechanisms once these restrictions were lifted [37, 38]. Moreover, unlike Hong Kong in where the control measures were lifted gradually over eight months, Beijing completed the transition within only one month. A modelling study supported that, compared to gradually removing control measures, removing major COVID-19 control measures within a short period would lead to a larger increase in the number of contacts between people [39]. These variations in policy changes might contribute to the differences in behavioral changes among GBMSM between Beijing and Hong Kong. In mainland China, the government was more likely to use enforcement strategies, such as executive orders and regulations, to implement COVID-19 preventive measures. The implementation of these measures was closely monitored by government staff. In contrast, the implementation of COVID-19 preventive measures mainly depends on the compliance of residents in Hong Kong. Therefore, it is understandable that the impact of these measures on the behaviors of GBMSM during the pandemic and the magnitude of behavioral changes after these measures were lifted were larger in Beijing.

The correlations between self-reported changes in other HIV-related behaviors and increase in CAS were similar between GBMSM in Beijing and Hong Kong. Self-reported increases in the frequency of anal sex with RP and NRP were associated with an increase in the frequency of CAS. Given the high and increasing prevalence of CAS after entering the post-pandemic era, it is expected that an increase in the number of anal sex would lead to more episodes of CAS. A self-reported increase in the frequency of SDU was also associated with an increase in CAS. Psychoactive substances used for SDU would adversely affect users’ capacity to perceive and respond to risks during sexual sex, leading to CAS [40]. Similar to the findings of previous studies, self-reported increase in the use of social and sexual networking applications to seek male partners was associated with an increase in CAS in this study [10, 41]. Moreover, self-reported increases in the frequency of using HIV testing, PrEP and other HIV prevention services were also correlated with an increase in CAS. GBMSM with an increased frequency of CAS might perceive a higher risk of HIV transmission, and hence a stronger motivation to use these HIV prevention services.

Perceived increases in the overall risk of HIV and COVID-19 transmission were associated with increase in CAS among GBMSM in both cities. Such findings suggested that GBMSM were aware of the risk of HIV and COVID-19 transmission through sexual risk behaviors. However, GBMSM in Beijing or Hong Kong might become less concerned about COVID-19 overtime due to the protection conferred by natural infection and COVID-19 vaccination. Therefore, it is not necessary to highlight the risk of COVID-19 through CAS in future programs. Although similar proportions of GBMSM in Beijing and Hong Kong perceived an increased risk of COVID-19, more GBMSM in Beijing had an increased fear of the disease. An increase in fear was associated with a self-reported increase in CAS among GBMSM in Beijing but not among GBMSM in Hong Kong. In mainland China (including Beijing), a massive outbreak of COVID-19 occurred soon after the relaxation of control measures in December 2022 [42]. It has been estimated that over 75% of Chinese people were infected within 2 weeks [43]. Difficulties in accessing COVID-19 testing and medical care further aggravate the fear of COVID-19. In contrast, most Hong Kong people contracted COVID-19 in early 2022, and the “resume normal” did not cause another wave of outbreak [26, 27]. Such a difference in the COVID-19 situation might partially explain why GBMSM in Hong Kong were less afraid of COVID-19. It is alarming that some GBMSM reported a decrease in self-efficacy of consistent condom use during anal sex in the post-pandemic era, especially among GBMSM in Beijing. Enhancing self-efficacy might be a useful strategy to reduce CAS among GBMSM in both cities, as a perceived decrease in self-efficacy was associated with a self-reported increase in CAS. Enhancement of self-control skills is warranted and rehearsals to persuade sex partners to use condoms consistently may be useful components of future health promotion programs.

Our study has several implications for HIV prevention service planning in the post-pandemic era. First, clustering of risk behaviors (i.e., CAS, anal sex with non-regular male sex partners, and SDU) was observed among GBMSM in both cities. Hence, there is a need to improve HIV prevention through a holistic approach that addresses the shared determinants of various risk behaviors. Second, given the association between frequency of online partner seeking through dating applications and the increase in CAS, it is possible to make use of these dating applications to access GBMSM at higher risk of HIV infection and deliver targeted interventions in the future. There have been successful examples of using BlueD and Grindr to promote HIV testing and safe sex behaviors for GBMSM [44, 45]. In addition, it is necessary to improve GBMSM’s access to HIV testing, PrEP and other HIV prevention services, as GBMSM at higher risk had higher motivation to seek these services. One-stop sexual health and HIV prevention service could make it easier for GBMSM to access needed services and support by reducing the number of locations they need to travel to [46].

This study had some limitations. First, similar to previous studies, participants were recruited by convenience sampling through multiple sources [10, 21, 25]. HIV-positive GBMSM were under-sampled in both cities [3–5]. Therefore, the samples might not represent GBMSM in Beijing or Hong Kong. Previous studies have shown that disruption in HIV treatment services caused by COVID-19 and its control measures have negative impact on treatment initiation and adherence [47]. People living with HIV also had fewer connections with their sex partners, and experienced prolonged isolation, stress, and anxiety during the pandemic [48]. However, there was a lack of studies investigating post-pandemic changes in sexual behaviors and HIV-related services utilization among HIV-positive GBMSM. It is possible that HIV treatment initiation and adherence would increase after treatment service resumes normal in the post-pandemic era among HIV-positive GBMSM. Moreover, the lifting of COVID-19 control measures may have a similar impact on the sexual behaviors among GBMSM of different HIV sero-status. Respondent-driven sampling might be a better sampling strategy in future studies [49]. Second, we were unable to collect information from GBMSM who refused to participate in the study. The characteristics of refusals and participants might be different. Selection bias existed. However, the impact of selection bias might be limited as the response rate was relatively high. Third, using self-reported data was another major limitation of this study. Participants might have under-reported sexual risk behaviors and over-reported HIV prevention service utilization due to social desirability bias. Such bias might have led to an under-estimation of the increase in sexual risk behaviors and over-estimation of the increase in HIV service utilization in this study. The potential social desirability bias could have been minimized by having trained and experienced interviewers conduct the interviews and by ensuring the anonymity of study participation. Fourth, we measured self-reported changes in behaviors and perceptions. Recall bias existed. An observational longitudinal study is a better design for understanding the changes in HIV-related behaviors and perceptions among GBMSM after entering the post-pandemic era. However, there was a lack of such longitudinal studies among GBMSM in China. Moreover, the cross-sectional study design could not establish causal relationships. Lastly, since this was an exploratory study, the assumptions used to plan for the sample size were arbitrary.

## Conclusions

Sexual risk behaviors were prevalent among GBMSM in Beijing and Hong Kong in the post-pandemic era. More GBMSM in Beijing than their counterparts in Hong Kong reported increases in the frequency of sexual risk behaviors (e.g., CAS, anal sex with NRP, and SDU) and HIV prevention services comparing their current situation with the time during COVID-19. Self-reported increase in the frequency of CAS was associated with increases in other sexual risk behaviors and HIV-related service utilization among participants in both cities. There are hence needs for improving HIV prevention through a holistic approach that addresses the shared determinants of various risk behaviors. One-stop sexual health and HIV prevention service should be considered, which could make it easier for GBMSM to access the necessary services and support.

## Data Availability

No datasets were generated or analysed during the current study.

## Abbreviations

GBMSM: Gay bisexual and other men who have sex with men
COVID-19: Coronavirus disease 2019.
CAS: Condomless anal sex.
SDU: Sexualized drug use.
PrEP: Pre-exposure prophylaxis.
RP: Regular male sex partners.
NRP: Non-regular male sex partners.
OR: Crude odds ratio.
AOR: Adjusted odds ratios.
CI: Confidence interval.
